# 252 Moving with nature: developing guidelines to promote physical activity in nature for those living with mental health problems

**DOI:** 10.1093/eurpub/ckae114.276

**Published:** 2024-09-26

**Authors:** Paul Sellars, Alexis Bennett, Diane Crone, Jenny Mercer, Debbie Clayton

**Affiliations:** Centre for Health, Activity and Wellbeing Research, Cardiff Metropolitan University, Wales, United Kingdom; Centre for Health, Activity and Wellbeing Research, Cardiff Metropolitan University, Wales, United Kingdom; Centre for Health, Activity and Wellbeing Research, Cardiff Metropolitan University, Wales, United Kingdom; Centre for Health, Activity and Wellbeing Research, Cardiff Metropolitan University, Wales, United Kingdom; Centre for Health, Activity and Wellbeing Research, Cardiff Metropolitan University, Wales, United Kingdom

**Keywords:** physical activity, nature, wellbeing, mental health

## Abstract

**Purpose:**

Mental illness is strongly associated with physical inactivity. Subsequently those individuals with mental health problems are at an increased risk of premature death due to non-communicable diseases (e.g., cardiovascular diseases and diabetes). Therefore, interventions which aim to improve both the physical and mental health of those with mental health problems are crucial. One such approach is nature-based activities (e.g., ecotherapy). Generally, nature-based interventions are considered an effective, low cost, and complementary intervention, to promote positive health and wellbeing. However, even with nature-based activities becoming more evident in health promotion, particularly through social prescribing, it remains an emergent area which requires further research to better understand the ways in which physical activity in nature can support those with mental health problems. Thus, the current research seeks to develop practical guidelines to support the use of nature-based programs in promoting the physical and mental health of those living with mental health problems.

**Methods:**

To achieve the project aim two objectives are sought, first, a scoping review with a narrative synthesis will be conducted to bring together exist recommendations/applied implications concerning physical activity in nature for enhanced mental health and wellbeing. Next, through a three stage Delphi methodology the existing recommendations/applied implications will be further developed into a set of practical guidelines. Participants in the Delphi will be selected based upon their expertise and knowledge of physical activity in nature, coming from academic and applied practice backgrounds.

**Results:**

The scoping review found 918 results, 55 of which were submitted to full-text screening, with 18 qualifying for the review. Following narrative synthesis, 49 recommendations/applied implications were found and subsequently developed into 10 practical guidelines concerning: person-centred, natural environment, activity type, programme duration, session duration, intensity, social interaction, achievement/learning, environmental responsibility, and target populations. Currently, preparations for the Delphi methodology are ongoing.

**Conclusion:**

Overall, through a scoping review and subsequent narrative synthesis, 10 practical guidelines concerning physical activity in nature for enhanced mental health and wellbeing​ were developed.

